# Association between Dietary Inflammatory Index and stroke prevalence among American adults: NHANES 2007 to 2016

**DOI:** 10.1097/MD.0000000000049198

**Published:** 2026-06-12

**Authors:** Chunchao Lian, Ping Zhong, Mingshuai Song, Zhenyu Wei

**Affiliations:** a Shidong Hospital Affiliated to University of Shanghai for Science and Technology, Shanghai, China.

**Keywords:** cross-sectional study, Dietary Inflammatory Index, logistic regression, NHANES, stroke

## Abstract

Limited evidence links chronic inflammation to stroke prevalence, and the Dietary Inflammatory Index (DII) serves as a valid tool to quantify the inflammatory potential of diets. This cross-sectional study utilized data from the National Health and Nutrition Examination Survey 2007 to 2016, enrolling 27,250 adult participants. DII was calculated based on 24 dietary components and categorized into tertiles (Q1: anti-inflammatory diet, Q2: intermediate diet, Q3: pro-inflammatory diet). Stroke status was determined by participants’ self-report of physician diagnosis, and covariates included demographic characteristics, socioeconomic status, lifestyle factors, and comorbidities. Weighted logistic regression models and restricted cubic spline analysis were employed to explore the association between DII and stroke. Among the participants, 981 (3.6%) had a history of stroke, with the mean DII significantly higher in stroke patients than in non-stroke patients (mean ± standard deviation: 1.65 ± 1.91 vs 0.96 ± 2.06, *P* < .001). In the minimally adjusted Model 2, as the DII grouping level increased, the odds of stroke prevalence also increased (odds ratio: 1.80, 95% confidence interval: 1.23, 1.91, *P* < .001). Restricted cubic spline analysis revealed a linear association between DII and stroke (nonlinear *P* = .5016), and subgroup analysis indicated significant interactions of this association with age and educational attainment (*P* < .05). In conclusion, a pro-inflammatory diet is positively associated with stroke prevalence in American adults, with a linear and stable relationship across different subgroups. Optimizing dietary inflammatory potential may be associated with lower stroke prevalence.

## 1. Introduction

Stroke is a globally significant threat to human health, caused by multiple factors that induce cerebrovascular damage and subsequent focal or generalized brain tissue impairment. According to the latest data from the Global Burden of Disease Study 2021, stroke causes approximately 7 million deaths annually.^[1]^

In recent years, a growing body of evidence has indicated that stroke is associated with systemic inflammation, including infectious and noninfectious inflammation, which plays an important role in the pathogenesis and progression of stroke.^[2]^ Elevated levels of inflammatory mediators such as interleukin-6 (IL-6), C-reactive protein, and lipoprotein-associated phospholipase A are linked to an increased risk of stroke.^[3,4]^ The elevation of circulating inflammatory biomarkers (e.g., interleukin-1β, IL-6, and tumor necrosis factor-α [TNF-α]) induced by abnormal immune activation is closely associated with the onset of stroke.^[4]^ Studies have found that soluble lectin-like oxidized low-density lipoprotein receptor-1 is an inflammation-induced lipid receptor that is not only closely correlated with atherosclerosis but also associated with stroke risk.^[5,6]^ A mounting body of evidence supports the association between elevated inflammatory biomarkers and an increased risk of stroke, stroke recurrence, poststroke vascular events, and mortality.^[7–10]^ It has been confirmed that anti-inflammatory agents, such as statins, can reduce the risk of stroke.^[11]^ This also reflects the fact that a chronic low-grade inflammatory state is a key event in stroke susceptibility.

Among numerous modifiable factors associated with higher odds of stroke, dietary patterns are closely associated with stroke risk by regulating the inflammatory status.^[12,13]^ Studies have shown that the Western dietary pattern is positively correlated with plasma concentrations of inflammatory markers, while healthy dietary patterns are associated with lower inflammatory levels.^[14]^ To quantify the pro-inflammatory/anti-inflammatory potential of diets, the Dietary Inflammatory Index (DII) was first proposed by Cavicchia et al in 2009 and further optimized by Shivappa et al in 2014.^[15,16]^ Its core logic is to integrate the inflammatory effects of 24 dietary components to obtain a total score reflecting the overall inflammatory potential of the diet.

Although the association between DII and stroke has received preliminary attention, existing systematic studies remain limited, particularly lacking epidemiological evidence in large multiracial populations. In view of this, the present cross-sectional study was conducted using data from the National Health and Nutrition Examination Survey (NHANES) 2007 to 2016, aiming to explore the association between DII and stroke prevalence. By analyzing the potential association patterns between the two across different racial backgrounds, this study intends to provide new epidemiological evidence for strategies related to lower odds of stroke and lay a foundation for understanding the population-specific mechanisms underlying the role of dietary inflammatory potential in stroke pathogenesis.

## 2. Methods

### 2.1. Study population

**Figure 1.**
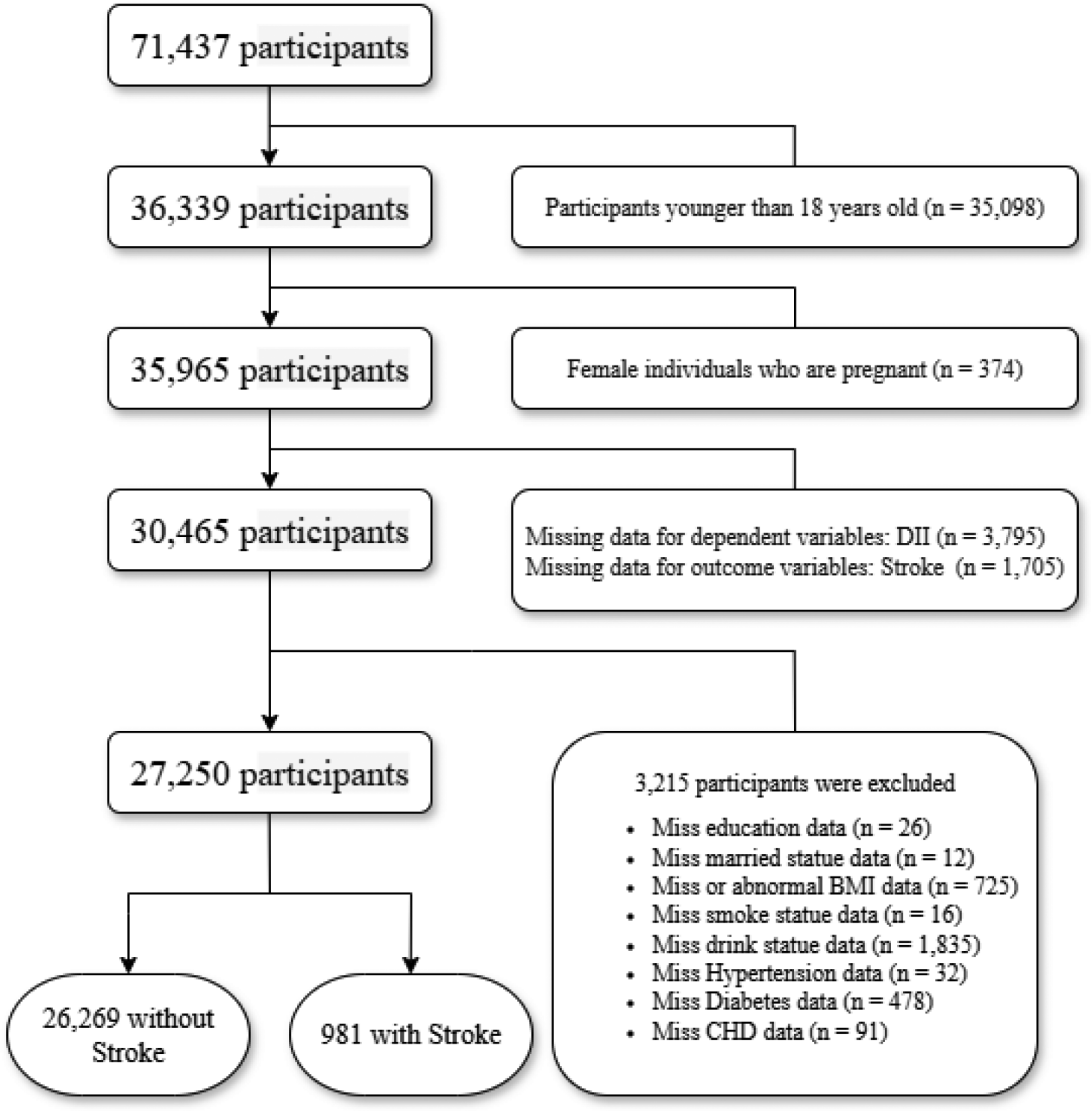
Flowchart of the inclusion of participants. CHD = coronary heart disease, DII = Dietary Inflammatory Index.

Data for this study were derived from the NHANES 2007 to 2016 dataset, a survey jointly conducted by the US Centers for Disease Control and Prevention and the National Center for Health Statistics. Employing a multistage sampling design, the sample is representative of the US population. The NHANES study protocol was approved by the National Center for Health Statistics Institutional Review Board, and all participants provided written informed consent. This cross-sectional study initially enrolled 71,437 participants, who were screened based on the following criteria: exclusion of individuals younger than 18 years (n = 35,098), pregnant females (n = 374), participants with missing or invalid DII data (n = 3795), and those with missing stroke status data (n = 1705). Additionally, 3215 participants were excluded due to missing or abnormal data for covariates, including education level, marital status, body mass index (BMI), smoking status, alcohol consumption, hypertension, diabetes mellitus, and coronary heart disease (CHD). Ultimately, 27,250 participants were included in the final analysis. Figure 1 illustrates in detail the flowchart of participant inclusion and exclusion.

### 2.2. DII

Dietary data in NHANES were collected during visits to the Mobile Examination Center using the first reliable 24-hour dietary recall method to record participants’ dietary intake. DII in this study was calculated based on 24 dietary components available in the database, including protein, carbohydrates, cholesterol, total saturated fatty acids, total monounsaturated fatty acids, total polyunsaturated fatty acids, vitamin A, vitamin B1, vitamin B2, vitamin B6, vitamin B12, vitamin C, vitamin D, vitamin E, niacin, folate, β-carotene, iron, magnesium, zinc, selenium, alcohol, caffeine, and dietary fiber. The calculation of DII followed a standardized process:


$$\begin{array}{cc} & \begin{array}{c} Z-score=\left(\left(daily\;mean\;intake-global\;daily\;mean\;intake\right)/standard\;deviation\right)\\ Z-score\prime =Z-score\to \left(converted\;to\;a\;percentile\;score\right)\;\times 2-1 \end{array}\;\\ & DII=\sum Z-score\prime \;\times \;the\;inflammatory\;ef\;fect\;\\ & score\;of\;each\;dietary\;component \end{array}$$

Participants were divided into 3 groups according to tertiles of DII scores: Q1 (DII < 0.04, anti-inflammatory diet group), Q2 (0.04 ≤ DII < 2.07, intermediate diet group), and Q3 (DII ≥ 2.07, pro-inflammatory diet group), with Q1 serving as the reference group.

### 2.3. Diagnosis of stroke

Stroke was used as the outcome variable in this study, and participants’ stroke status was ascertained from the NHANES medical conditions questionnaire. Participants who responded “Yes” to the question “Has a doctor or other health professional ever told you that you have had a stroke?” were classified as having a history of stroke; all others were classified as having no stroke history. As with all self-reported health conditions, this definition is susceptible to recall bias and potential misclassification, which may introduce some degree of measurement error into the outcome assessment. Furthermore, the NHANES database does not distinguish between ischemic and hemorrhagic stroke subtypes, so specific associations according to stroke type could not be examined in this analysis.

### 2.4. Information on other covariates

To control for potential confounding effects, the following covariates were collected and included in the study, categorized and defined in accordance with database field definitions and relevant literature standards: demographic characteristics (age [continuous variable, ≥18 years], gender [male/female], race/ethnicity [non-Hispanic White/non-Hispanic Black/Mexican American/Other Hispanic/Other races]), socioeconomic status (education level [<high school/high school graduate or equivalent/college or above], marital status [married/living with a partner/never married/widowed/divorced/separated]), lifestyle factors (smoking status [never smoked: <100 cigarettes in a lifetime/former smoker: ≥100 cigarettes in a lifetime but not currently smoking/current smoker: ≥100 cigarettes in a lifetime and smoking daily]^^[17]^^; alcohol consumption [never drank: <12 drinks in a lifetime/former drinker: <12 drinks in the past year but ≥12 drinks in a lifetime/current drinker: ≥12 drinks in the past year]); BMI (continuous variable, calculated as weight [kg] divided by height [m] squared), comorbidities (CHD [self-reported physician diagnosis]; hypertension [self-reported physician diagnosis or mean systolic blood pressure ≥ 140 mm Hg and/or mean diastolic blood pressure ≥ 90 mm Hg]; diabetes mellitus [self-reported physician diagnosis or glycated hemoglobin ≥ 6.5% or fasting blood glucose ≥ 7.0 mmol/L or random blood glucose ≥ 11.1 mmol/L]; dyslipidemia [high-density lipoprotein cholesterol <50 mg/dL for females or <40 mg/dL for males, or low-density lipoprotein cholesterol ≥ 130 mg/dL, or triglycerides ≥ 150 mg/dL, or total cholesterol ≥ 200 mg/dL, or current use of cholesterol-lowering medications]).

### 2.5. Statistical analysis

All statistical analyses were performed using RStudio (R version 4.4.1, R Foundation for Statistical Computing). Data were weighted using sampling weights provided by NHANES to ensure national representativeness. Continuous variables conforming to a normal distribution were expressed as “mean ± standard deviation,” and categorical variables were presented as “frequency (percentage).” Intergroup comparisons were conducted using *t* tests for continuous variables and chi-square tests for categorical variables.

A directed acyclic graph-based causal framework was used for covariate selection. Sociodemographic and lifestyle factors were treated as confounders, whereas hypertension, diabetes, CHD, and dyslipidemia were considered potential mediators on the pathway from DII to stroke. Adjustment for mediators may bias results toward the null. Three models were therefore constructed: a crude model with no adjustment, a minimally adjusted model, and a fully adjusted model. The fully adjusted model was not regarded as representing the true total effect. Weighted logistic regression models were used to analyze the association between DII and stroke, with 3 models constructed: Model 1 (no covariate adjustment); Model 2 (adjusted for age, gender, race/ethnicity, education level, marital status, smoking status, alcohol consumption, and BMI); and Model 3 (further adjusted for CHD, hypertension, diabetes mellitus, and dyslipidemia based on Model 2). Given that these comorbidities may lie on the causal pathway as potential mediators, Model 3 was not interpreted as estimating the total causal effect. Wald tests were performed for trend analysis to evaluate the dose-response relationship between DII tertiles and stroke prevalence.

Restricted cubic spline (RCS) curves were applied to evaluate the linearity of the association between DII and stroke prevalence, using the minimally adjusted model (adjusted for sociodemographic and lifestyle factors only). Subgroup analyses were conducted to explore the modifying effects of factors such as age, gender, and race/ethnicity on the association, with interaction effects tested using weighted logistic regression. Subgroup analyses were performed across all included covariates to explore potential effect modification. Sensitivity analyses were performed using multiple imputation to handle missing covariate data, and the aforementioned weighted logistic regression analyses were repeated on the imputed data to verify the stability of the results. In addition, implausible energy reporters were excluded using gender-specific thresholds (males: <800 or >4200 kcal/d; females: <500 or >3500 kcal/d^[18]^), and the main analyses were repeated in the remaining valid sample. A two-tailed *P*-value < .05 was considered statistically significant.

## 3. Results

### 3.1. Baseline characteristics

A total of 27,250 participants were included in the analysis, with a weighted mean age of 47.52 years. The overall stroke prevalence among all participants was 3.6%, and the weighted mean DII score was 0.98. Compared with non-stroke patients, stroke patients were more likely to be older, non-Hispanic Black, less educated, and current or former smokers. Among comorbidities, the proportions of stroke patients with diabetes mellitus, hypertension, and dyslipidemia were relatively higher. Table 1 presents detailed baseline characteristics of all participants stratified by stroke status. The mean DII score in the stroke group was significantly higher than that in the non-stroke group (1.65 vs 0.96, *P* < .001).

**Table 1:** Baseline characteristics of all participants.

Characteristic	Overall (N = 27,250)	Stroke		*P*-value
		Yes (N = 981)	No (N = 26,269)	
Gender, n (%)				.4
Female	13,534 (50.42)	486 (52.44)	13,048 (50.37)	
Male	13,716 (49.58)	495 (47.56)	13,221 (49.63)	
Age (yr), mean (SD)	47.52 ± 16.79	63.78 ± 14.22	47.08 ± 16.64	<.001
Race, n (%)				<.001
Non-Hispanic Black	5896 (10.86)	276 (15.42)	5620 (10.74)	
Mexican American	3932 (8.06)	87 (4.86)	3845 (8.15)	
Non-Hispanic White	11,702 (68.77)	481 (68.02)	11,221 (68.79)	
Other Hispanic	2874 (5.52)	71 (3.43)	2803 (5.58)	
Other race	2846 (6.78)	66 (8.27)	2780 (6.74)	
Education, n (%)				<.001
<High school	6620 (15.88)	347 (28.93)	6273 (15.53)	
High school	6168 (21.90)	268 (27.76)	5900 (21.74)	
>High school	14,462 (62.22)	366 (43.32)	14,096 (62.73)	
Marital status, n (%)				<.001
Married/living with partner	16,035 (63.30)	492 (57.25)	15,543 (63.47)	
Never married	5195 (18.56)	85 (7.32)	5110 (18.87)	
Widowed/divorced/separated	6020 (18.13)	404 (35.43)	5616 (17.66)	
BMI, mean (SD)	29.02 ± 6.55	29.84 ± 6.74	28.99 ± 6.54	.020
Drink status, n (%)				<.001
Never	3921 (10.96)	165 (15.48)	3756 (10.84)	
Former	19,712 (78.05)	648 (69.59)	19,064 (78.28)	
Current	3609 (10.99)	167 (14.93)	3442 (10.88)	
Smoke status, n (%)				<.001
Never	15,042 (55.11)	374(38.76)	14,668 (55.55)	
Former	7637 (28.49)	389 (39.02)	7248 (28.20)	
Current	4571 (16.40)	218 (22.22)	4353 (16.24)	
CHD, n (%)				<.001
Yes	1097 (3.29)	164 (17.54)	933 (2.90)	
No	26,153 (96.71)	817 (82.46)	25,336 (97.10)	
Hypertension, n (%)				<.001
Yes	10,178 (33.02)	759 (73.94)	9419 (31.91)	
No	17,072 (66.98)	222 (26.06)	16,850 (68.09)	
Diabetes, n (%)				<.001
Yes	4767 (12.80)	382 (33.98)	4385 (12.23)	
No	22,483 (87.20)	599 (66.02)	21,884 (87.77)	
Dyslipidemia, n (%)				<.001
Yes	6492 (25.24)	270 (27.52)	6222 (23.69)	
No	20,758 (74.76)	711 (72.48)	20,047 (76.31)	
DII, mean (SD)	0.98 ± 2.06	1.65 ± 1.91	0.96 ± 2.06	<.001

BMI = body mass index, CHD = coronary heart disease, DII = Dietary Inflammatory Index, SD = standard deviation.

### 3.2. Relationship between DII and stroke

Univariate weighted logistic regression analysis revealed significant associations between stroke and variables including age, race/ethnicity, alcohol consumption, smoking status, CHD, hypertension, diabetes mellitus, and dyslipidemia (all *P* < .05; Table 2).

**Table 2:** Univariate weighted logistic regression analysis.

Characteristic	OR	95% CI	*P*-value
Age	1.07	1.06, 1.07	<.001
Gender			
Female	–	–	
Male	0.92	0.76, 1.12	.4
Race			
Non-Hispanic Black	–	–	
Mexican American	0.42	0.29, 0.59	<.001
Non-Hispanic White	0.69	0.59, 0.81	<.001
Other Hispanic	0.43	0.32, 0.57	<.001
Other race	0.85	0.58, 1.25	.4
Education			
<High school	–	–	
High school	0.37	0.29, 0.48	<.001
>High school	0.69	0.56, 0.84	<.001
Marital status			
Married/living with partner	–	–	
Never married	0.43	0.31, 0.59	<.001
Widowed/divorced/separated	2.22	1.81, 2.73	<.001
BMI	1.02	1.00, 1.03	.014
Drink status			
Never	–	–	
Former	0.62	0.47, 0.82	<.001
Current	0.96	0.72, 1.28	.8
Smoke status			
Never	–	–	
Former	1.98	1.65, 2.38	<.001
Current	1.96	1.51, 2.54	<.001
CHD	7.12	5.14, 9.85	<.001
Hypertension	6.05	4.93, 7.44	<.001
Diabetes	3.69	2.99, 4.56	<.001
Dyslipidemia	3.09	1.71, 5.59	<.001
DII.quantile	1.19	1.14, 1.24	<.001
Q1	–	–	
Q2	1.52	1.18, 1.97	.002
Q3	2.08	1.69, 2.56	<.001

BMI = body mass index, CHD = coronary heart disease, CI = confidence interval, DII = Dietary Inflammatory Index, OR = odds ratio.

In the multivariate weighted logistic regression analysis, DII was divided into tertiles, and 3 models were constructed to adjust for potential confounders. In Model 1 (without any covariate adjustment), compared with the anti-inflammatory diet group (Q1), the intermediate diet group (Q2) and pro-inflammatory diet group (Q3) had significantly increased stroke risk, with odds ratios (OR) of 1.52 (95% confidence interval [CI]: 1.18, 1.97, *P* = .002) and 2.08 (95% CI: 1.69, 2.56, *P* < .001), respectively. After adjusting for age, race/ethnicity, education level, smoking status, alcohol consumption, and BMI in Model 2, the results showed that higher DII tertiles were associated with higher odds of stroke. Specifically, compared with Q1, Q2 and Q3 were associated with 32% and 53% higher odds of stroke prevalence, respectively (OR = 1.32 (95% CI: 1.00, 1.76, *P* = .049) for Q2 and OR = 1.47 (95% CI: 1.18, 1.83, *P* < .001) for Q3).

These results indicate that even after excluding the impact of underlying comorbidities, a pro-inflammatory diet remained independently associated with higher odds of stroke (Table 3).

**Table 3:** Multivariate weighted logistic regression analysis.

DII.quantile	Model 1			Model 2			Model 3		
	OR	95% CI	*P*-value	OR	95% CI	*P*-value	OR	95% CI	*P*-value
Q1	–	–		–	–		–	–	
Q2	1.52	1.18, 1.97	.002	1.32	1.00, 1.74	.049	1.32	1.00, 1.76	.049
Q3	2.08	1.69, 2.56	<.001	1.53	1.23, 1.91	<.001	1.47	1.18, 1.83	<.001
Trend			<.001			<.001			<.001

Model 1: with no covariate adjustment.

Model 2: adjusted for age, gender, race/ethnicity, marital status, education, smoke status, and drink status.

Model 3: Model 2 + coronary heart disease, hypertension, diabetes, dyslipidemia.

CI = confidence interval, OR = odds ratio.

**Figure 2.**
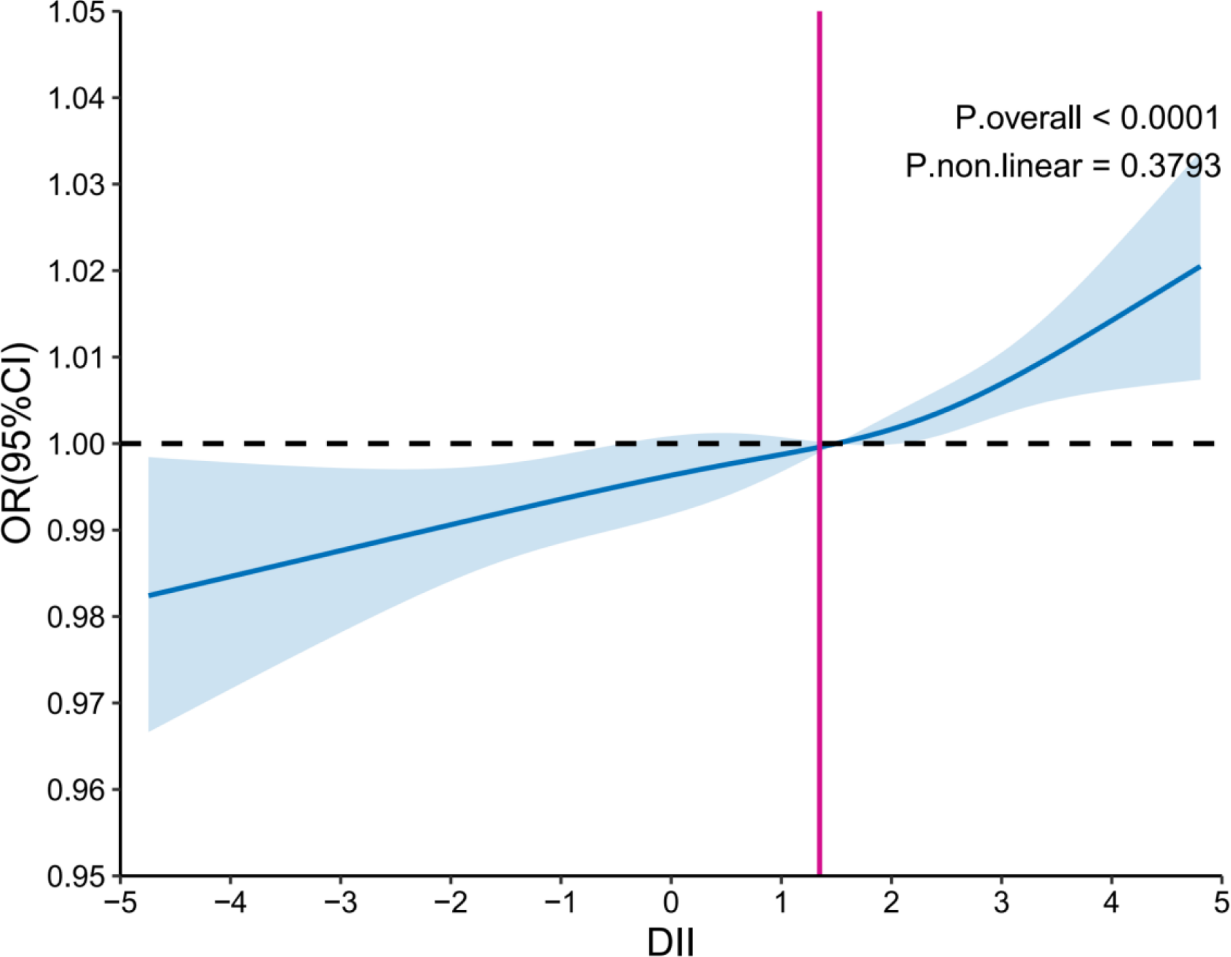
Association between DII and stroke odds ratio, weighted analysis. CI = confidence interval, DII = Dietary Inflammatory Index, OR = odds ratio.

To evaluate the dose-response association between DII and stroke prevalence, we examined linearity and conducted RCS analyses in Model 2. The results showed that with the continuous increase of DII level, the odds of stroke prevalence (*P* for nonlinear = .3794, *P* for overall < .001) gradually increased (Fig. 2).

### 3.3. Subgroup, sensitivity, and additional analyses

**Figure 3.**
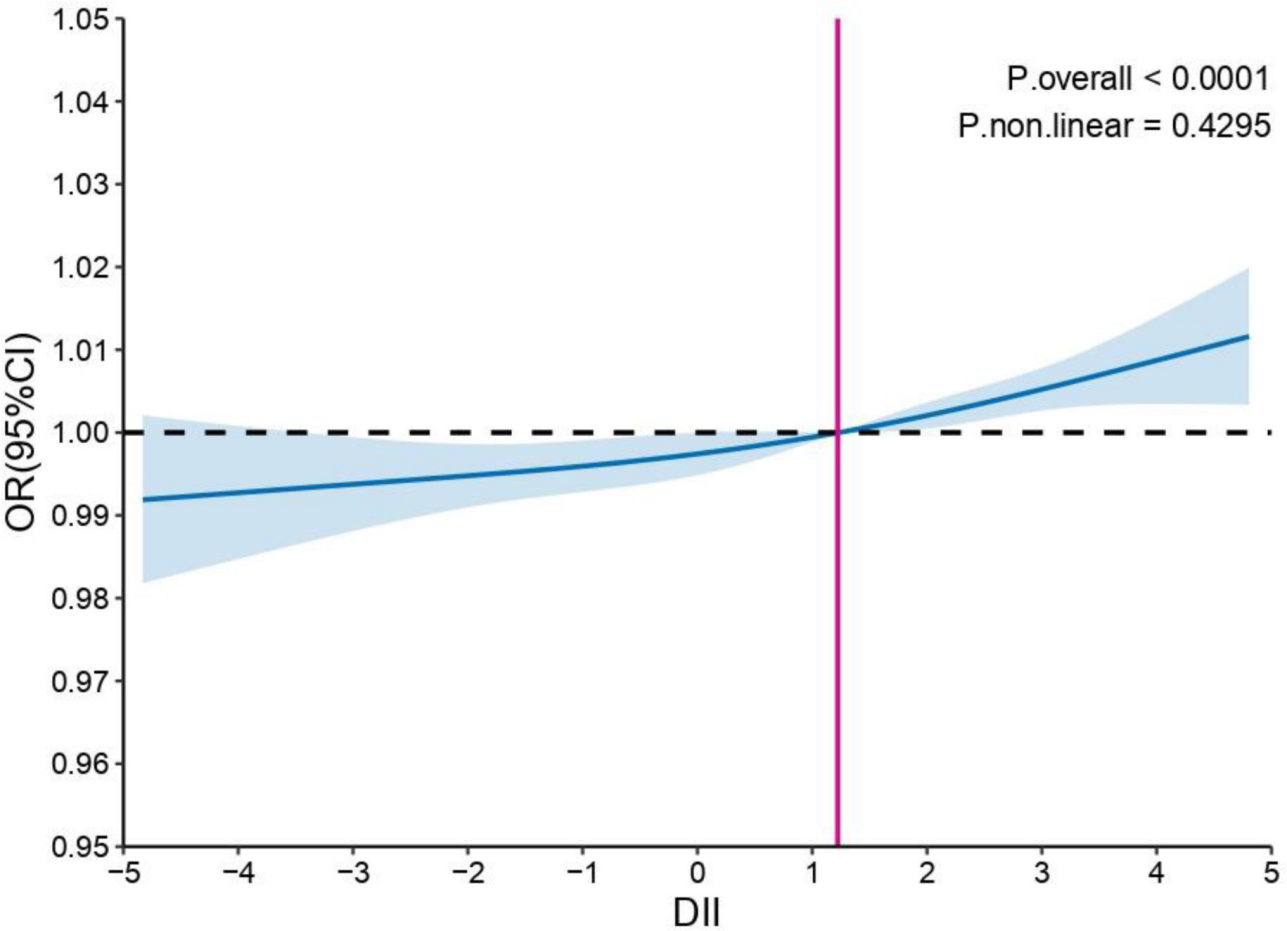
Association between DII and stroke odds ratio in individuals aged 60 years and younger, weighted analysis. CI = confidence interval, DII = Dietary Inflammatory Index, OR = odds ratio.

Subgroup analysis indicated that, based on the above risk factors, there was no evidence of effect modification or interaction except for age (*P* > .05; Fig. 3).

Meanwhile, covariates excluding comorbidities were selected and integrated into binary variables to further analyze their interaction with DII tertiles and stroke (Table 4). We found that the positive association between DII and stroke was generally stable across the study population (OR > 1). Age was the only factor that significantly modified the association between DII and stroke, with significant differences in the strength of association observed before and after 60 years of age (*P* for interaction = .01).

**Table 4:** Subgroup analysis of the association between DII and stroke, weighted.

Characteristic	OR 95% CI	*P*-value	Characteristic	OR 95% CI	*P*-value	*P*.for.interaction
Age, yr						.01
18–60			>60			
Q1	–	–	Q1	–	–	
Q2	1.99 (1.22, 3.23)	.006	Q2	1.21 (0.86, 1.71)	.3	
Q3	2.92 (1.81, 4.72)	<.001	Q3	1.48 (1.12, 1.96)	.007	
Trend test		<.001			.007	
Gender						.52
Female			Male			
Q1	–	–	Q1	–	–	
Q2	1.99 (1.27, 3.10)	.003	Q2	1.32 (0.97, 1.79)	.077	
Q3	2.66 (1.79, 3.95)	<.001	Q3	1.83 (1.37, 2.45)	<.001	
Trend test		<.001			<.001	
Drink status						.18
Yes			No			
Q1	–	–	Q1	–	–	
Q2	1.54 (1.10, 2.08)	.012	Q2	1.51 (1.03, 2.22)	.012	
Q3	1.95 (1.48, 2.57)	<.001	Q3	2.19 (1.48, 2.57)	<.001	
Trend test		<.001			<.001	
Smoke status						.99
Yes			No			
Q1	–	–	Q1	–	–	
Q2	1.51 (1.10, 2.08)	.012	Q2	1.51 (1.03, 2.22)	.012	
Q3	1.95 (1.48, 2.57)	<.001	Q3	2.19 (1.49, 3.22)	<.001	
Trend test		.007			<.001	
BMI						.05
<24			≥24			
Q1	–	–	Q1	–	–	
Q2	1.92 (1.01, 3.63)	.046	Q2	1.42 (1.04, 1.95)	.028	
Q3	2.25 (1.31, 3.87)	.004	Q3	2.02 (1.56, 2.61)	<.001	
Trend test		.004			<.001	

BMI = body mass index, CI = confidence interval, DII = Dietary Inflammatory Index, OR = odds ratio.

**Figure 4.**
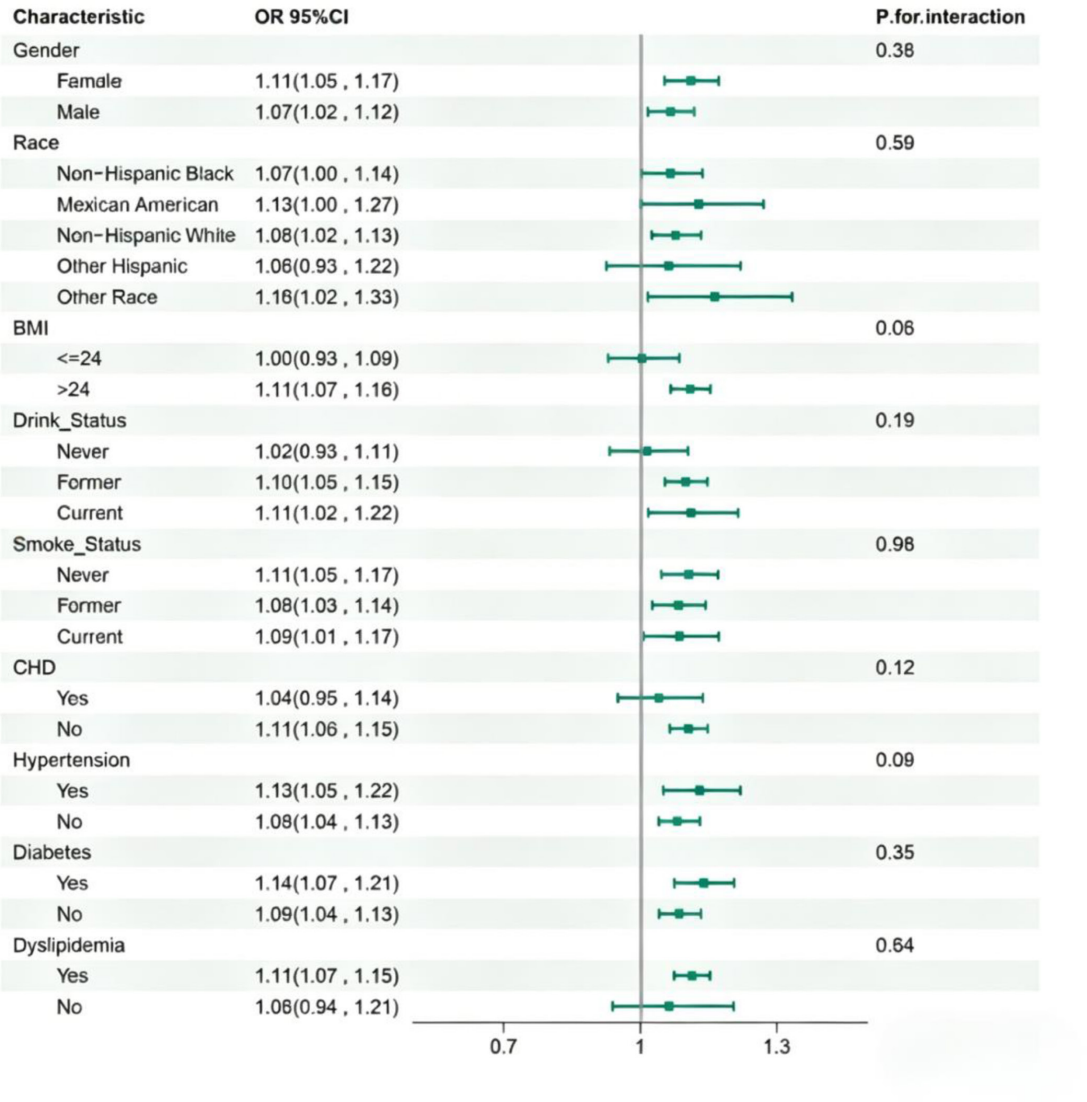
Association between the DII and stroke according to basic features. BMI = body mass index, CHD = coronary heart disease, CI = confidence interval, DII = Dietary Inflammatory Index, OR = odds ratio.

In addition, due to the strong interaction with age, RCS curves were plotted for stroke cases with early onset. According to previous literature, the incidence and risk drivers of stroke differ significantly before and after 60 years of age, which is a natural cutoff for dividing risk stages.^[19]^ RCS analysis indicated a linear association between DII score and stroke in this age group (*P* for nonlinearity = .4295, *P* for overall < .001), with no apparent inflection point in risk observed. This indicates that even in young populations, a slight increase in dietary pro-inflammatory potential was associated with higher odds of stroke, which is consistent with the overall study results (Fig. 4).

For sensitivity analysis, multiple imputation was used to handle missing covariate data, and an optimal set was selected for multivariate logistic regression analysis. The results of the multivariate analysis were consistent with those obtained after excluding individuals with missing covariates (Table 5).

**Table 5:** Association between DII and stroke, after multiple interpolation of missing values, weighted.

Characteristic	OR	95% CI	*P*-value
DII			
Q1	–	–	
Q2	1.34	1.12, 1.61	.002
Q3	1.86	1.61, 2.16	<.001

CI = confidence interval, DII = Dietary Inflammatory Index, OR = odds ratio.

Additionally, the association between DII and stroke risk was further verified after excluding outliers of energy intake. The results were consistent with those of the DII analysis without excluding energy intake outliers, confirming the robustness of the positive association between dietary inflammatory potential and stroke prevalence (Table 6).

**Table 6:** Association between DII and stroke risk, after excluding outliers of energy intake.

Characteristic	OR	95% CI	*P*-value
DII			
Q1	–	–	
Q2	1.46	1.12, 1.90	.006
Q3	2.01	1.59, 2.54	<.001

CI = confidence interval, DII = Dietary Inflammatory Index, OR = odds ratio.

## 4. Discussion

According to our study, in this comprehensive retrospective cross-sectional study utilizing the large-scale NHANES dataset from 2007 to 2016, a total of 27,250 participants were enrolled, and 24 dietary components were selected for the calculation of the DII. Data analysis revealed a positive correlation between DII levels and stroke prevalence. Notably, RCS analysis indicated a linear relationship between DII levels and stroke risk (nonlinear *P* < .05). After adjusting for confounding factors, including age, race/ethnicity, education level, smoking status, alcohol consumption, BMI, CHD, hypertension, diabetes mellitus, and dyslipidemia, the correlation between DII levels and stroke risk remained statistically significant. Subgroup analysis confirmed the stability of this correlation across various subgroups. These findings hold important clinical implications.

Stroke is associated with a complex array of risk factors, and unhealthy dietary patterns are well-established modifiable triggers for stroke.^[20]^ The DII serves not only as a key indicator for assessing the risk of chronic low-grade inflammation but also as a quantitative tool for evaluating dietary patterns. The Western dietary pattern is a typical pro-inflammatory dietary model, characterized by high DII scores (indicating strong pro-inflammatory potential) and corresponding high intakes of refined carbohydrates, sugar, trans fats, and ultra-processed foods.^[21]^ In contrast, the Mediterranean dietary pattern, which is rich in anti-inflammatory components from core foods (olive oil, fatty fish, fruits, and vegetables), generally yields low DII scores (indicating strong anti-inflammatory potential). Individuals with pro-inflammatory diets have a significantly increased risk of developing hypertension, diabetes mellitus, CHD, and dyslipidemia,^[22–24]^ which is consistent with the findings of our study.

Currently, DII has been confirmed to be correlated with circulating inflammatory markers, including interleukin-1β, IL-6, interleukin-10, TNF-α, and C-reactive protein, and is widely used to assess the overall pro-inflammatory and anti-inflammatory properties of an individual’s diet.^[25]^ Pro-inflammatory cytokines such as IL-6 and TNF-α induced by high pro-inflammatory diets can disrupt the vascular endothelial barrier and accelerate the formation of atherosclerotic plaques, which is directly linked to the “plaque rupture–thrombosis” mechanism of ischemic stroke.^[26,27]^ Meanwhile, pro-inflammatory diets may elevate blood pressure (a core inducementof hemorrhagic stroke) by activating the sympathetic nervous system and the renin-angiotensin-aldosterone system, and promote a hypercoagulable state by increasing fibrinogen levels.^[22,28]^ However, direct data demonstrating the link between DII and stroke through these 2 mechanisms are still lacking.

This study has several limitations. First, as a cross-sectional study, we cannot establish a causal relationship between DII and stroke, and reverse causation is highly plausible – individuals with a history of stroke may have modified their dietary habits after diagnosis, which could lower DII scores and distort or weaken the observed cross-sectional association. Second, DII was derived from a single 24-hour dietary recall, which suffers from recall bias and cannot reflect long-term dietary habits. Importantly, significant day-to-day dietary variability exists in the general population, causing marked fluctuations in the intake of DII-related dietary components across days and introducing random non-differential measurement error into DII assessment. This error can attenuate the observed association between DII and stroke risk by pushing the effect estimate toward the null and may even distort the true dose-response relationship between dietary inflammatory potential and stroke prevalence when dietary fluctuations are substantial. Third, stroke status was based on self-report in response to the question, “Has a doctor or other health professional ever told you that you have had a stroke?” Self-reported stroke in NHANES has imperfect validity and may include transient ischemic attack or misclassified events. In addition, NHANES data do not allow for distinguishing between hemorrhagic and ischemic stroke subtypes, which may limit the interpretability of the findings. Fourth, we conducted a number of subgroup analyses to explore potential differences in the relationship between DII and stroke. All subgroup variables were selected in advance based on previous studies and clinical importance. However, performing many subgroup tests may increase the chance of false-positive results. Therefore, the findings from subgroup analyses should be interpreted cautiously. Fifth, the study population was predominantly non-Hispanic White (accounting for 68.77%). Other racial groups may have differences in dietary structures, health social determinants (such as healthcare accessibility and health awareness), and genetic backgrounds, which may limit the generalizability of the results. Therefore, a study adopting a prospective cohort design, incorporating repeated dietary assessments (such as multiple 24-hour dietary recalls and food frequency questionnaires), and expanding racial diversity (such as including East Asian populations with high stroke incidence) would be more conducive to verifying these results and establishing a causal relationship.

## 5. Discussion

The present study demonstrated a positive association between the DII and stroke prevalence. These findings highlight the importance of evaluating dietary inflammatory potential as a marker for stroke. Future large-scale prospective studies are warranted to confirm these results and elucidate the causal relationship between pro-inflammatory diets and stroke.

## Author contributions

**Conceptualization:** Chunchao Lian, Ping Zhong.

**Methodology:** Mingshuai Song, Zhenyu Wei.

**Formal analysis:** Mingshuai Song.

**Investigation:** Zhenyu Wei.

**Software:** Zhenyu Wei.

**Data curation:** Chunchao Lian.

**Project administration:** Ping Zhong, Mingshuai Song, Zhenyu Wei.

**Resources:** Ping Zhong, Mingshuai Song, Zhenyu Wei.

**Supervision:** Ping Zhong, Mingshuai Song, Zhenyu Wei.

**Writing – original draft:** Chunchao Lian, Zhenyu Wei.

**Writing – review & editing:** Ping Zhong, Zhenyu Wei.
